# Usage of a Localised Microflow Device to Show that Mitochondrial Networks Are Not Extensive in Skeletal Muscle Fibres

**DOI:** 10.1371/journal.pone.0108601

**Published:** 2014-09-26

**Authors:** Joseph Bruton, Gavin D. M. Jeffries, Håkan Westerblad

**Affiliations:** 1 Department of Physiology and Pharmacology, Karolinska Institute, Stockholm, Sweden; 2 Department of Chemical and Biological Engineering, Chalmers University of Technology, Gothenburg, Sweden; Cinvestav-IPN, Mexico

## Abstract

In cells, such as neurones and immune cells, mitochondria can form dynamic and extensive networks that change over the minute timescale. In contrast, mitochondria in adult mammalian skeletal muscle fibres show little motility over several hours. Here, we use a novel three channelled microflow device, the multifunctional pipette, to test whether mitochondria in mouse skeletal muscle connect to each other. The central channel in the pipette delivers compounds to a restricted region of the sarcolemma, typically 30 µm in diameter. Two channels on either side of the central channel use suction to create a hydrodynamically confined flow zone and remove compounds completely from the bulk solution to internal waste compartments. Compounds were delivered locally to the end or side of single adult mouse skeletal muscle fibres to test whether changes in mitochondrial membrane potential were transmitted to more distant located mitochondria. Mitochondrial membrane potential was monitored with tetramethylrhodamine ethyl ester (TMRE). Cytosolic free [Ca^2+^] was monitored with fluo-3. A pulse of carbonyl cyanide 4-(trifluoromethoxy) phenylhydrazone (FCCP, 100 µM) applied to a small area of the muscle fibre (30 µm in diameter) produced a rapid decrease in the mitochondrial TMRE signal (indicative of depolarization) to 38% of its initial value. After washout of FCCP, the TMRE signal partially recovered. At distances greater than 50 µm away from the site of FCCP application, the mitochondrial TMRE signal was unchanged. Similar results were observed when two sites along the fibre were pulsed sequentially with FCCP. After a pulse of FCCP, cytosolic [Ca^2+^] was unchanged and fibres contracted in response to electrical stimulation. In conclusion, our results indicate that extensive networks of interconnected mitochondria do not exist in skeletal muscle. Furthermore, the limited and reversible effects of targeted FCCP application with the multifunctional pipette highlight its advantages over bulk application of compounds to isolated cells.

## Introduction

In mammalian cells, mitochondria exist in a variety of forms from the almost universal picture of an ovoid structure not more than one µm long seen in cells ranging in size from hepatocytes to neurones to the long thread-like branching structures attaining a length of 50 µm or longer found in human fibroblasts [1], [2]. The spatiotemporal distribution of mitochondria is not fixed in most cell types but frequently, large numbers are found close to sites of high metabolic demand [1], [3]. In some cells, mitochondria are dynamic organelles that change their shape and develop protrusions within minutes even in the absence of any external stimulus [4]–[6]. Mitochondria in organisms as diverse as fungi and mice can also adapt quickly to metabolic disturbances within the cell. For example, in adult neurones and immature or foetal cardiomyocytes, mitochondria can reversibly elongate and fuse under hypoxic and other stressful conditions [3], [7]. Dynamic formation of mitochondria to mitochondria connections has been demonstrated in cells as diverse as cortical neurones [1] and fibroblasts [2]. These connections can form and break quite readily and allow diffusion of fluorescent labels between distant mitochondrial areas which suggest that the mitochondrial matrix has a uniform internal ionic and protein solution [8]–[12].

In adult skeletal and cardiac muscle, it is unclear as to whether functional networks of mitochondria exist and how extensive mitochondrial interconnections are [13]–[15]. The densely arranged filaments of actin and myosin in muscle dedicated to contraction, which make up more than 80% of the cell volume in skeletal muscle, leaves little free space for mitochondria to move around. Mitochondrial localization in mammalian fibres is similar in oxidative fatigue-resistant and glycolytic fatigue-sensitive muscle fibres. Indeed, the majority of mitochondria in mammalian skeletal muscle are located in pairs on the *z*-line side of t-tubules (e.g.[16]–[18]). High resolution electron microscopy studies on rat and human skeletal muscle studies show that addition to this repeating paired pattern, additional subsarcolemmal and longitudinal columns of mitochondria are frequently present. The longitudinal and, to a lesser extent, the subsarcolemmal columns show branches extending both to other adjacent mitochondria and transversely for up to 7 µm over several myofibrils [19]–[21]. In skeletal muscle, mitochondria showed no discernible movement or change in shape over a period of several hours in mouse muscle fibres that were fatigued or subjected to mild osmotic shock [13], [22], [23]. However, the finding that the number of mitochondria increases following periods of endurance training indicates that fission and linkage between mitochondria can occur [24], [25]. Furthermore, the fusion or disappearance of mitochondria that occurs after muscles have been immobilised also indicates that mitochondrial structure is adaptable [26].

To date, it has been relatively difficult to test the hypothesis that mitochondria are arranged in networks in skeletal muscle. Here, we have taken the approach of using a novel microflow-pipette device to deliver chemicals to small discrete regions (typically 30 µm in diameter) of muscle cells. The device was used to deliver chemicals, including the uncoupling agent FCCP, which depolarises mitochondria, to the end or side of adult muscle fibres while measuring changes in mitochondrial membrane potential close to and away from the region of depolarised mitochondria. The main result of these studies indicates that there is limited coupling between mitochondria in skeletal muscle since only mitochondria close to the site of application of FCCP are depolarised while distant mitochondria remain polarised.

## Materials and Methods

### Animals

Adult female mice (C57BL/6; weight 30 g) were killed by rapid neck disarticulation, and flexor digitorum brevis (FDB) muscles were removed. The experiments were approved by the Stockholm North local ethical committee (Ethical permission number N152/11).

### Enzymatic isolation of adult muscle fibres

FDB muscles were placed in Tyrode solution and cleaned of tendons, connective tissue and blood vessels using jeweller’s forceps and a micro-iris scissor. The vast majority of fibres in the FDB muscles are type IIa/x [27] and are approximately 600 to 800 µm long. The cleaned muscles were incubated at 37°C for 2–3 hours in 0.3% collagenase type I (Sigma) in DMEM (Invitrogen) supplemented with 10% foetal bovine serum (Invitrogen). At the end of the incubation period, muscles were transferred to 3 ml of DMEM and triturated to dissociate individual muscle fibres. Approximately 300 µl of the resultant suspension of muscle fibres was placed on laminin (Sigma) coated glass-bottom Petri dishes (Mattek) and allowed to attach for five minutes: Finally, a further 3 ml DMEM supplemented with antibiotic, antimycotic solution (1 µl/ml, Sigma) was added to the dishes. Dishes were placed in an incubator at 37°C for use on the following day.

### Solutions

Muscle fibres were superfused with Tyrode solution containing (mM): 121 NaCl, 5 KCl, 1.8 CaCl_2_, 0.5 MgCl_2_, 0.4 NaH_2_PO_4_, 0.1 EDTA, 24 NaHCO_3_, and 5.5 glucose. Solutions were bubbled with 5% CO_2_/95% O_2_ (pH 7.4). Fresh solutions of 100 µM carbonyl cyanide 4-(trifluoromethoxy) phenylhydrazone (FCCP) or 50 mM H_2_O_2_, were prepared each morning for use in the microflow pipette device. All experiments were carried out at room temperature (22–24°C).

### Confocal microscopy and indicators

Mitochondrial membrane potential was monitored using tetramethylrhodamine ethyl ester (TMRE, Invitrogen). Mitochondria were loaded by exposing muscle fibres to 0.1 µM TMRE for 10 min and washing for a further 20 min. Mitochondrial reactive oxygen species (ROS) production was measured using MitoSOX Red which is oxidized by superoxide but not easily by H_2_O_2_ or other ROS -generating systems (Invitrogen). Fibres were exposed to 4 µM MitoSOX Red for 15 min and thereafter washed for 20 min. Free cytosolic [Ca^2+^] ([Ca^2+^]_i_) was measured in the dissociated FDB fibres using the fluorescent Ca^2+^ indicator fluo-3 (Invitrogen). Fibres were exposed to fluo-3 AM (4 µM) for 20 min, followed by 20 min washing. In all experiments, only those fibres which gave a vigorous twitch response to a 1 ms electrical stimulus after loading of the indicators were used.

Fibres were imaged using a Bio-Rad MRC 1024 confocal unit fitted with a dual Calypso laser (Cobolt, Solna, Sweden) attached to a Nikon Diaphot 200 inverted microscope and a Nikon Plan Apo 20x dry objective, (N.A. 0.75 Nikon, Tokyo, Japan). A few experiments were carried out with a Plan Fluor 40x oil objective (N.A. 1.3 Nikon, Tokyo, Japan). Excitation wavelengths used were 491 nm (fluo-3) and 531 nm (MitoSOX Red and TMRE), and the emitted light was collected through appropriate filters for the various indicators (522 nm for fluo-3, 585 or 605 nm for MitoSOX Red and TMRE, respectively). Images were analysed with the image analysis programme Fiji [28]. Fluorescence intensity of the indicators is expressed as a ratio, F/F_0_, where F represents the fluorescence intensity at each time point and F_0_ is the fluorescence at the start of the experiment.

### Microflow pipette device

The characteristics of the unique microflow device, the multifunctional pipette (developed by Avalance Biotech AB, Sweden) have been described in detail elsewhere [29]. The most important characteristic of this pipette is the localised delivery coupled with its temporal resolution. This enables targeting of a test solution to a small surface area of a cell without any significant leakage of the test solution to the bulk solution surrounding the fibres. In the experiments reported here, the typical region of the muscle fibre exposed to a test solution was 30 µm in diameter. In some experiments, the area of the fibre exposed to the dye was increased to 60 or even 100 µm.

**Figure 1:**
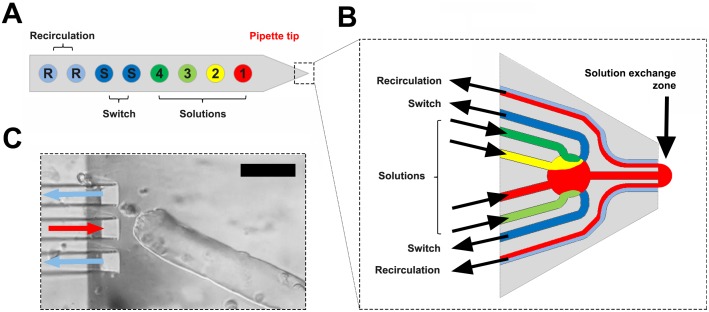
Schematic diagram of the multifunctional pipette used in the experiments. **A.** Cartoon of the general layout with four wells (Solutions, numbered 1 to 4) which can store separate chemicals for delivery through a single channel in the pipette tip and two wells (Switch) which store the waste sucked through the pair of channels on the side of the pipette tip. **B.** The key elements in the pipette tip shown in more detail with a solution exiting through the central channel and removed through the paired vacuum suction channels to the side of the central channel. **C.** View of the pipette placed close to the end of a muscle fibre during an actual experiment. The red and blue arrows indicate delivery and removal of solution that define the delivery zone and the end of the fibre sits in front of the central channel. Black calibration bar in the upper right of **C** represents 75 µm.

The solution flow from the tip of the pipette is defined by modulating the pressure in a delivery channel and in a pair of suction vacuum channels that lie on either side of the delivery channel (Figure 1). Delivery of a defined cone of solution was monitored by spiking a Tyrode solution with the cell-impermeant dye, sulforhodamine B, which has no deleterious effects on muscle fibres [22]. During experiments, images were obtained at either 5 or 7 s intervals. The first two images were always control images without solution delivered from the pipette, the second two were images where a sulforhodamine B solution was delivered from the pipette. Next, two images were obtained where only Tyrode was delivered and actively removed by the pipette ensuring that fresh Tyrode solution surrounded the fibre. Images were then obtained continuously while the test compound was delivered (usually 2 minutes). In most experiments, further images were obtained after the compound had been removed to observe recovery of the targeted region or possible contamination effects.

### Statistics

Values are given as means ± S.E.M (n) where n is the number of separate observations. Statistically significant differences (p<0.01) were determined using a Student’s t-test.

## Results

### Effects of FCCP application with multifunctional pipette on mitochondrial TMRE fluorescence

**Figure 2:**
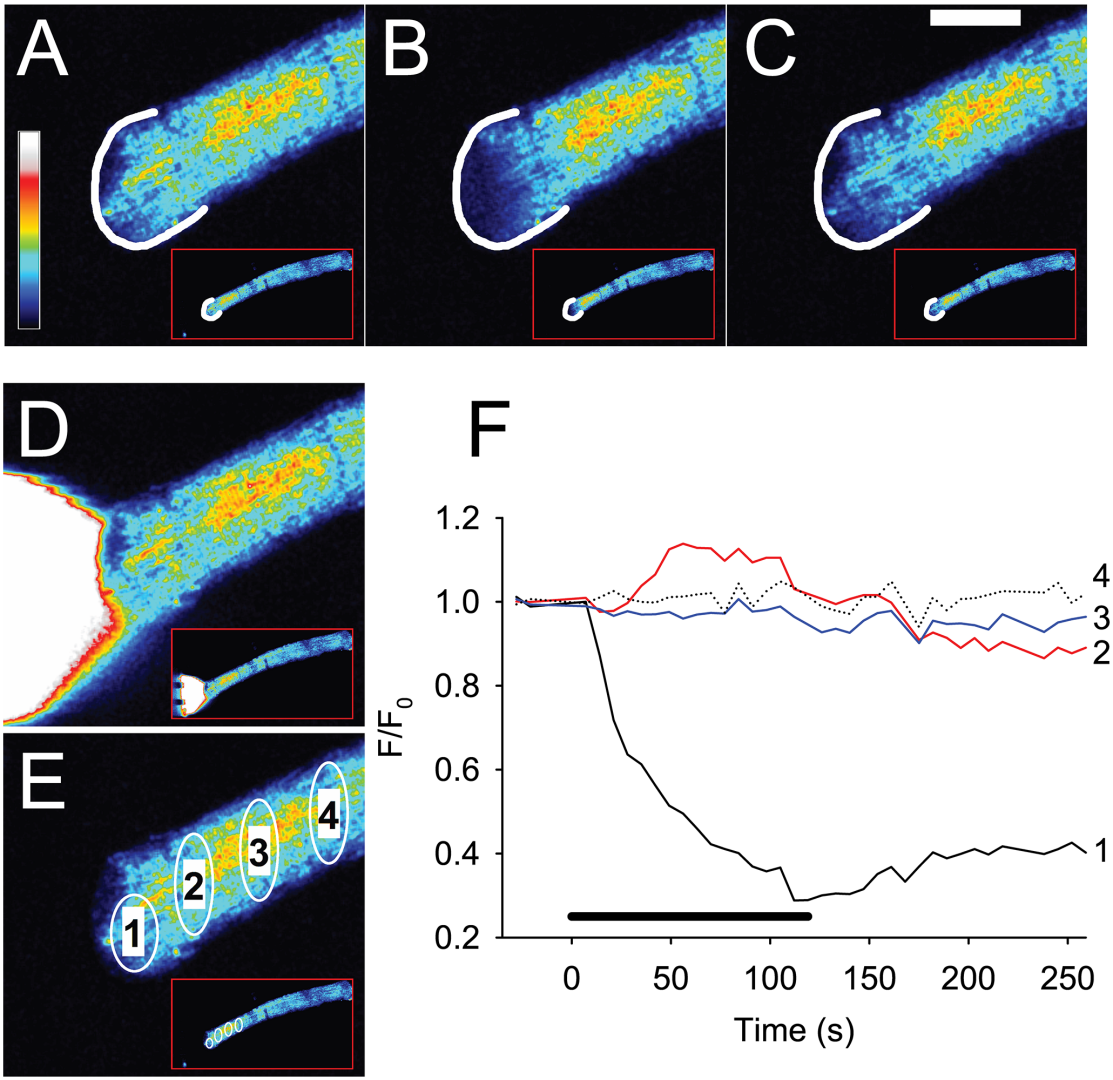
Application of 100 µM FCCP to the end of a FDB fibre induces localised release of TMRE from mitochondria indicating that these have become depolarised. The colour panels **A–C** show the TMRE signal in the end of the magnified fibre and also in the whole FDB fibre in the lower right of each panel (marked by red box) at rest (**A**), at the end of 120 s of FCCP application (**B**) and 130 s after removal of FCCP (**C**). In panels **A–C,** a white semi-circle indicates the end of the fibre. Note that loss of mitochondrial TMRE occurs at the site of FCCP application, spreads less than 50 µm distally and is partially reversible. Panel **D** shows a white cone visualised using sulforhodamine B which indicates the region of the fibre exposed to FCCP. Panel **E** shows the sites at which changes in the TMRE signal were measured (numbered 1 to 4) during the experiment. The graph in panel **F** shows that in mitochondria close to the site of FCCP application, the TMRE signal falls to a minimum of ∼0.3 after 110–120 s. In contrast, at site 2 that is only 40 µm from the site of FCCP application, the TMRE signal actually increases during the period of FCCP application. At sites 100 and 150 µm distant from the site of FCCP application, there is no change in the TMRE signal. The coloured bar to the left in **A** shows the LUT from low (blue) to high (white) values applied to each pixel in the image. The thick black bar in **F** indicates the period of FCCP application. White horizontal calibration bar in **C** represents 50 µm.

Figure 2 and Movie S1 show the effect of a 120 s pulse of 100 µM FCCP applied to the end of a single dissociated FDB muscle fibre. In the front 10 µm of the fibre closest to the site of FCCP application, the mitochondrial TMRE signal (F/F_0_) decreased to a minimum of 0.3 of its initial value after 100 s (black trace 1 in Figure 2F). When FCCP application was stopped, there was a slight recovery in F/F_0_. Similar results were seen in a further 7 fibres. The maximum decrease in F/F_0_ during the period of exposure to FCCP was −0.62±0.04 and the recovery of the TMRE signal was 0.20±0.03 (n = 8). At distances of 100 µm and greater, F/F_0_ remained stable throughout the period of FCCP application (traces 3 and 4 in Figure 2). Interestingly, the TMRE released from the mitochondria closest to the FCCP pulse appeared to be taken up by neighbouring mitochondria such that there was an increase in F/F_0_ at about 50 µm away from the front of the FCCP pulse (trace 2 in Figure 2F).

**Figure 3:**
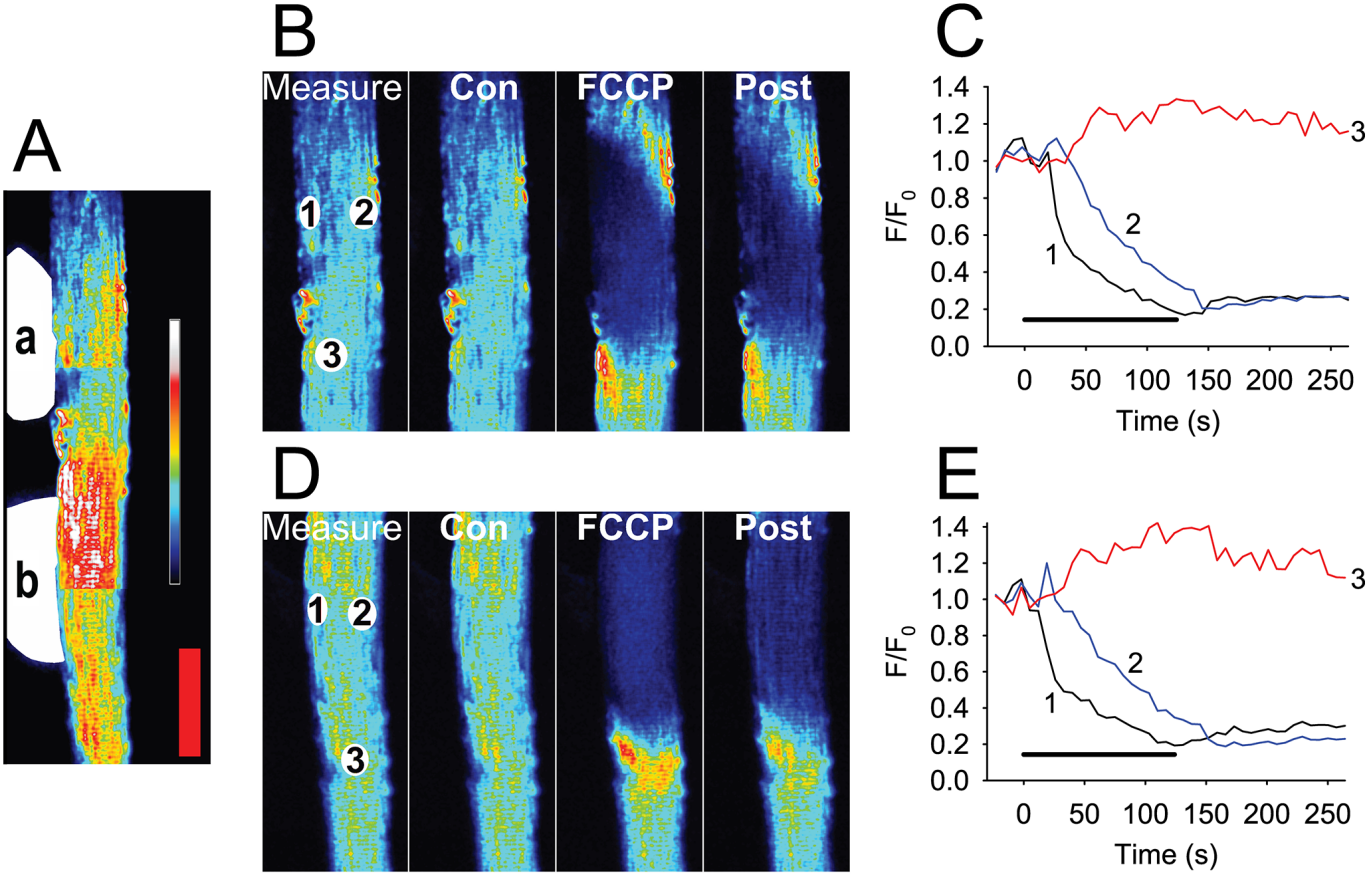
Sequential broadside application of 100 µM FCCP at two separate regions in the same FDB fibre. FCCP is applied sequentially to two regions of a fibre (**A,** indicated by the white areas labelled **a** and **b** to the left of the fibre) and induces release of TMRE from mitochondria only in the area exposed to FCCP. Panel **B** shows an enlargement of region **a** and panel **D** shows an enlargement of region **b**. Regions of interest (ROI 1 to 3) used to extract intensity traces for **C** and **E** are shown in **B**_measure_ and **D**_measure_ at rest (Con), at the end of 120 s of FCCP (FCCP) and 130 s after removal of FCCP (Post). Panels **C** and **E** are plots of the changes in the TMRE F/F_0_ at the three ROI sites before, during and after the application of FCCP to the fibre. In mitochondria at site 1, F/F_0_ falls to a minimum of ∼0.3 after 110–120 s. At site 2, F/F_0_ also declines with a time lag of about 20 s. Importantly at site 3 that is only 50 µm from the site of FCCP application, the TMRE signal does not decrease but rather increases during and after the period of FCCP application. The coloured bar to the right in **A** shows the LUT from low (blue) to high (white) values applied to each pixel in the image. Red vertical calibration bar in **A** represents 50 µm.

In further experiments, FCCP was applied to the side of 17 fibres to expose a larger area of the fibre to FCCP (Figure 3 and Movie S2). The results with broadside application of FCCP were similar to those found when FCCP was applied to the end of the fibre. The TMRE signal was lost at the site of FCCP application but at distances of 50 µm or further from the site of FCCP application, the TMRE signal remained high. In all the fibres studied, the maximum decrease in F/F_0_ during the period of exposure to FCCP was −0.64±0.03 and the recovery of the TMRE signal was 0.17±0.03 (n = 17). In a subset of the theses fibres, FCCP was applied to two sites along the side of the fibre. A typical example is shown in Figure 3 and in Movie S2. At both sites, there was a loss of the TMRE signal at the site of FCCP application (maximum decline at site 1 was −0.68±0.06 and the subsequent maximum decline at site 2 was −0.65±0.04, n = 8). In all fibres examined, all mitochondria at the site of FCCP application showed a loss of FCCP signal. However in mitochondria away from this area, the TMRE signal did not decline during the FCCP application. It is interesting to note that in mitochondria close to the sarcolemma and also those in the centre of the fibre, where mitochondrial interconnectivity would be expected to be greater [19], [21], the decrease in F/F_0_ and the rate of depolarization were similar. No creeping depolarization of the mitochondria, neither in the inter-myofibrillar mitochondria at the centre of the muscle fibre nor in the subsarcolemmal mitochondria was observed (e.g. see image sequence from 1 min 24 s to 1 min 59 s in Movie S2). Thus, focal application of FCCP to the end or side of a skeletal muscle fibre caused only a local decrease in the mitochondrial TMRE signal indicating that there were limited connections between the mitochondria either close to the sarcolemma or in the inter-myofibrillar space in the centre of the muscle fibres.

### Effects of FCCP application with multifunctional pipette on Ca^2+^ homeostasis

**Figure 4:**
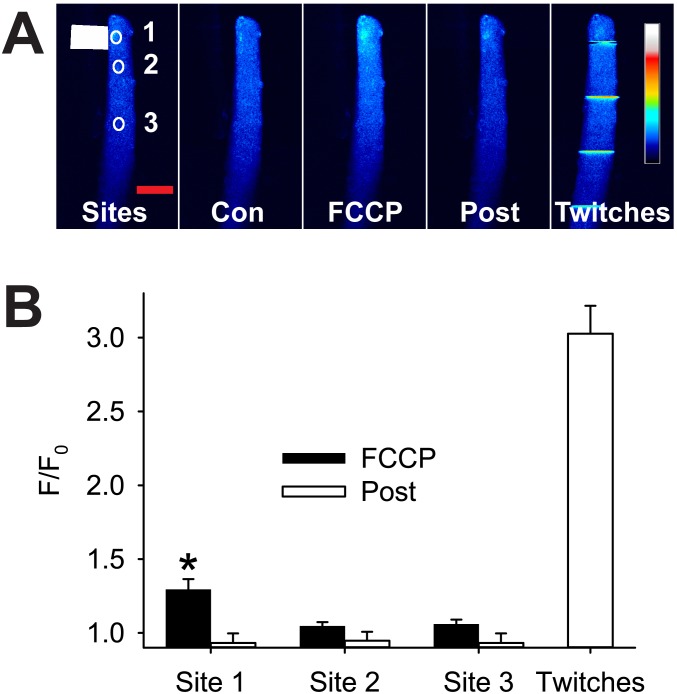
Local application of FCCP causes a transient increase in resting cytosolic [Ca^2+^]. Panel **A** shows typical fluo-3 fluorescence before (Con), during (FCCP) and after (Post) focal application of 100 µM FCCP (Sites, white rectangle) and twitch contractions evoked in the same fibre after FCCP (Twitches) in a single FDB muscle fibre. Note that FCCP induced a clear increase in fluo-3 signal only at site 1 (FCCP) which reversed completely after washout of FCCP (Post). **B** is a graph of mean F/F_0_ at 90 to 120 s exposure to FCCP (black bars) and at 120 s after removal of the FCCP (white bars). Note that the increase in fluo-3 F/F_0_ caused by exposure to FCCP is small compared to the increase seen in response to electrically evoked responses (Twitches). The coloured bar to the right in **A**_Twitches_ shows the LUT from low (blue) to high (white) values applied to each pixel in the image. Values shown are mean ± sem (n = 9), * indicates significantly different from resting F/F_0_, p<0.01. Red horizontal calibration bar in **A**_Sites_ represents 50 µm.

We were interested to see if FCCP-induced depolarization of the mitochondrial membrane potential had any effect on Ca^2+^ homeostasis and the resting Ca^2+^ concentration ([Ca^2+^]_i_) in the FDB fibres. Figure 4 shows that application of 100 µM FCCP for 120 s caused a significant increase in fluo-3 fluorescence (F/F_0_) only in the region of the fibre close to the FCCP. At two sites away from the FCCP application (50 µm and >150 µm), the fluo-3 signal was unchanged during the exposure to FCCP. The maximum increase in fluo-3 F/F_0_ after 120 s in FCCP (0.29±0.07, n = 13) was small when compared to the increase in F/F_0_ measured in a twitch after removal of FCCP (2.03±0.19, n = 9). Two minutes after the removal of FCCP, the fluo-3 F/F_0_ had returned to its resting level in all parts of the muscle fibre (Figure 4B). These results indicate that depolarization of the mitochondria had limited and completely reversible effects on Ca^2+^ homeostasis in the FDB fibres.

### Effects of H_2_O_2_ application with multifunctional pipette on MitoSOX signal

**Figure 5:**
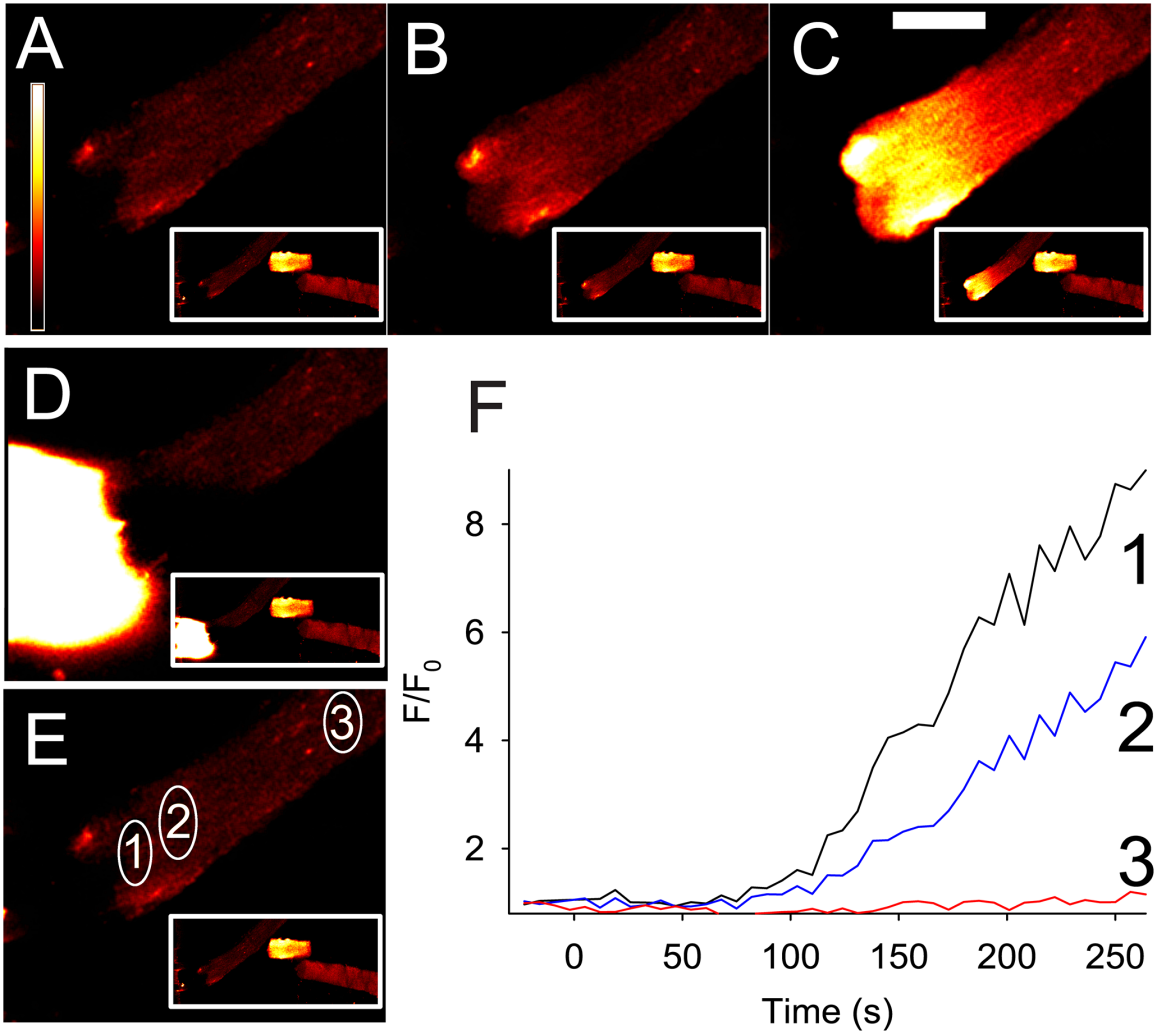
Typical effect of H_2_O_2_ application on the MitoSOX Red signal in an intact single FDB muscle fibre. Each panel **A–E** shows a magnification of the end of the fibre where H_2_O_2_ was applied and the white box in the lower right of each panel shows the whole length of the fibre. Importantly, in the two other fibres also seen in the white box, there is no change in the MitoSOX signal during the H_2_O_2_ application. **A** shows the FDB fibre at rest, and at two (**B**) and four minutes (**C**) after the start of H_2_O_2_ application. The coloured bar to the left in **A** shows the LUT from low (black) to high (white) values applied to each pixel in the image. **D** shows where the H_2_O_2_ was applied and **E** shows the sites where the MitoSOX signal was measured. The graph in **F** shows the time course of the changes in mitochondrial MitoSOX signal in the muscle fibre, H_2_O_2_ application was continuous from time 0. White horizontal calibration bar in **C** represents 50 µm.

An alternate way of detecting whether mitochondria form networks is to take advantage of the production of superoxide that occurs during mitochondrial respiration. Elimination of mitochondrial superoxide is impaired by the presence of H_2_O_2_ [30]. The MitoSOX Red signal (which is insensitive to H_2_O_2_) was measured during the focal application of H_2_O_2_ to the end of the fibre (Figure 5 and Movie S3). Over the first 50 s, the MitoSOX Red signal showed little change in any of the three sites (see Figure 5F) measured in the fibre. Thereafter, the signal increased markedly at the end of the fibre (Figure 5B–C, F). Conversely, away from the site of application of H_2_O_2_, the MitoSOX Red signal in the mitochondria showed only a modest change (see trace 3 in Figure 5F). Broadly similar results were found in a further five fibres and the mean increase in F/F_0_ at 4 min of H_2_O_2_ application was 3.60±0.90 at site 1, 2.19±0.62 at site 2 and 1.57±0.20 at site 3 (n = 6). The effects observed at site 3 are likely to be the result of a lengthy exposure to H_2_O_2_, which is a small molecule with rapid diffusivity. Once inside the fibre, the H_2_O_2_ could readily diffuse in the myoplasm to regions not under the direct flow zone defined by the microflow pipette, inhibiting superoxide elimination and hence creating an increase in the MitoSOX Red signal away from the region of application independent of any direct connections between mitochondria.

## Discussion

This study demonstrates that the multifunctional pipette can deliver minute amounts of a chemical to a small restricted area of a cell rapidly and reversibly. As the device is freestanding, disconnected from both the sample and the microscope, it can be used to exchange solutions in any experimental setup One caveat is that the cells need to either adhere natively or be attached to the dish surface using artificial means. Using this device we have shown here that delivery of the mitochondrial uncoupling agent, FCCP caused only mitochondria that lie directly under the flow path of FCCP to become depolarised. These mitochondria released the TMRE indicator which was then taken up by adjacent mitochondria. Even when relatively large areas of the fibre were exposed to the FCCP, mitochondrial depolarization was confined to those mitochondria underneath and very close to the site of FCCP application. Mitochondrial depolarisation was not detected in mitochondria that lay as little as 50 µm away from the border of the FCCP application. Mitochondria within this 50 µm zone may have been subjected to FCCP which was partially able to freely diffuse in the myoplasm. In addition, FCCP might have flowed through the short (<10 µm) branching structures that have been reported to link non-adjacent mitochondria in mammalian muscle [19]–[21]. However this mode must be limited given that the Movies S1 and S2 show that there is a clear border to the extent of mitochondrial depolarization during the FCCP application. An interesting point to note is that after FCCP application was stopped, there was a limited recovery of the mitochondrial TMRE signal. This is not surprising given that there is little free TMRE in the myoplasm of the fibre, since the TMRE released from the depolarised mitochondria during the FCCP application was either taken up by other mitochondria or diffused down the concentration gradient out of the cell.

Localised delivery of FCCP to either the end or side of a muscle fibre did not result in any major irreversible change in Ca^2+^ homeostasis in the muscle. The resting fluo-3 signal (reflecting [Ca^2+^]_i_) showed a modest increase during the period of FCCP exposure but rapidly returned to the resting level after FCCP was removed. The increase in fluo-3 fluorescence was most marked in the region of the fibre where mitochondrial depolarisation had occurred. Previous studies have shown that bathing muscle fibres in FCCP results in a marked increase in resting Ca^2+^ that is poorly reversed after washout of FCCP [31], [32]. The Ca^2+^ causing the increased [Ca^2+^]_i_ is unlikely to come from depolarised mitochondria since it was shown previously that mitochondrial [Ca^2+^] is low [33] and that mitochondria in mouse FDB muscle fibres do not accumulate Ca^2+^ [17]. A possible source of the rise is decreased activity of the Ca^2+^ ATPase in the sarcoplasmic reticulum membrane due to decreased availability of ATP. Regardless, the limited and reversible effects of the targeted application of FCCP with the microflow pipette highlight its advantages over bulk application of compounds to isolated cells.

Application of H_2_O_2_ caused only a modest change in the MitoSOX Red signal during the first two minutes in mitochondria located close to the site of application but thereafter the MitoSOX signal increased markedly. In contrast, the MitoSOX signal at sites about 150 µm further away showed no change during the entire period that H_2_O_2_ was applied. Again these results indicate that there is little communication between mitochondria in adult skeletal muscle fibres. In future experiments it would be interesting to use the microflow pipette on other cell types to see if the limited communication between mitochondria reported here in FDB fibres is specific to adult skeletal muscle fibres.

In conclusion, the present study used a novel microflow device, the multifunctional pipette, to deliver FCCP to localised areas of muscle fibres to examine the extent of mitochondrial connections. Our results show that communication between mitochondria in skeletal muscle fibres is limited.

## Supporting Information

Movie S1 **Local application of FCCP depolarises local but not distant mitochondria.** FCCP (100 µM) was applied with the microflow pipette to the end of a FDB fibre. An enlarged view of the fibre occupies the main area of the box and the whole fibre is shown enclosed in the red box at the lower right. Time refers to the time (in minutes and seconds) at which FCCP application started. The vertical bar is a LUT where black indicates low TMRE signal and white represents high TMRE. The bright white halo at times −00∶21 and −00∶14 is the delivery of a sulforhodamine B-spiked Tyrode solution indicating the area of the fibre exposed to the flow from the multifunctional pipette. White horizontal calibration bar represents 50 µm.
(AVI)

Movie S2 **Multiple local application of FCCP depolarises only local but not distant mitochondria.** FCCP (100 µM) was applied to two sites on the side of the same FDB fibre with the microflow pipette. An enlarged view of the fibre occupies the main area of the box and the whole fibre is shown enclosed in the red box on the right. Two timers are used. The first timer (white numbers) refers to the time (in minutes and seconds) at which FCCP application at the first site started while the second (blue numbers) refers to the time (in minutes and seconds) at which FCCP was applied to the second site. The vertical bar is a LUT where black indicates low TMRE signal and white represents high TMRE. The bright white halo shows the delivery of a sulforhodamine B-spiked Tyrode and indicates the area of the fibre exposed to the flow from the multifunctional pipette.
(AVI)

Movie S3 **Local application of H_2_O_2_ results in a localised mitochondrial increase in MitoSOX Red signal.** H_2_O_2_ (50 mM) was applied to the end of a FDB fibre with the microflow pipette. An enlarged view of the fibre occupies the main area of the box and the whole fibre together with two other fibres is shown enclosed in the red box at the lower right. Time refers to the time (in minutes and seconds) at which H_2_O_2_ application started. The vertical bar is a LUT where black indicates low MitoSOX signal and white represents a high MitoSOX signal. The bright white halo shows the delivery of a sulforhodamine B-spiked Tyrode to indicate the area of the fibre exposed to the flow from the multifunctional pipette.
(AVI)
